# Supplementary material for: Uncovering miRNA-mRNA Regulatory Modules in Developing Xylem of Pinus massoniana via Small RNA and Degradome Sequencing
Source: Int J Mol Sci. 2021 Sep 21;22(18):10154. doi: 10.3390/ijms221810154 (PMC8472836; doi:10.3390/ijms221810154)
Supplement: Supplementary file 1 [file ijms-22-10154-s001.zip › ijms-1365557-supplementary/Additional file S2.zip!/Additional file 2/novel_2_Cluster-18410.68235_292_TPlot.pdf]

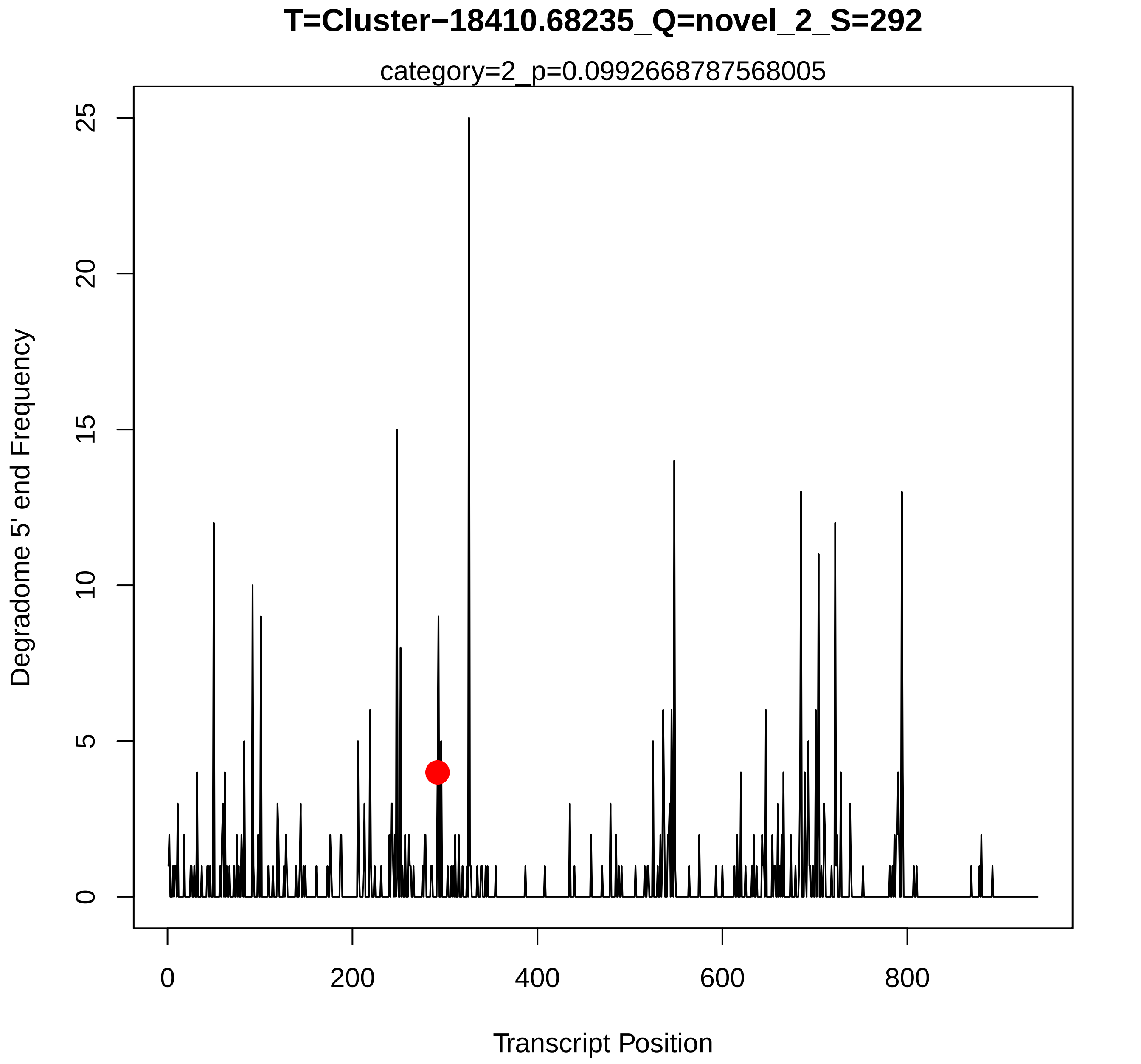
T=Cluster−18410.68235_Q=novel_2_S=292
category=2_p=0.0992668787568005
Degradome 5' end Frequency
Transcript Position
0
5
10
15
20
25
0
200
400
600
800
